# Risk Prediction of Emergency Department Visits in Patients With Lung Cancer Using Machine Learning: Retrospective Observational Study

**DOI:** 10.2196/53058

**Published:** 2023-12-06

**Authors:** Ah Ra Lee, Hojoon Park, Aram Yoo, Seok Kim, Leonard Sunwoo, Sooyoung Yoo

**Affiliations:** 1 Office of eHealth Research and Business Seoul National University Bundang Hospital Seongnam-si Republic of Korea; 2 Department of Radiology Seoul National University Bundang Hospital Seongnam-si Republic of Korea

**Keywords:** emergency department, lung cancer, risk prediction, machine learning, common data model, emergency, hospitalization, hospitalizations, lung, cancer, oncology, lungs, pulmonary, respiratory, predict, prediction, predictions, predictive, algorithm, algorithms, risk, risks, model, models

## Abstract

**Background:**

Patients with lung cancer are among the most frequent visitors to emergency departments due to cancer-related problems, and the prognosis for those who seek emergency care is dismal. Given that patients with lung cancer frequently visit health care facilities for treatment or follow-up, the ability to predict emergency department visits based on clinical information gleaned from their routine visits would enhance hospital resource utilization and patient outcomes.

**Objective:**

This study proposed a machine learning–based prediction model to identify risk factors for emergency department visits by patients with lung cancer.

**Methods:**

This was a retrospective observational study of patients with lung cancer diagnosed at Seoul National University Bundang Hospital, a tertiary general hospital in South Korea, between January 2010 and December 2017. The primary outcome was an emergency department visit within 30 days of an outpatient visit. This study developed a machine learning–based prediction model using a common data model. In addition, the importance of features that influenced the decision-making of the model output was analyzed to identify significant clinical factors.

**Results:**

The model with the best performance demonstrated an area under the receiver operating characteristic curve of 0.73 in its ability to predict the attendance of patients with lung cancer in emergency departments. The frequency of recent visits to the emergency department and several laboratory test results that are typically collected during cancer treatment follow-up visits were revealed as influencing factors for the model output.

**Conclusions:**

This study developed a machine learning–based risk prediction model using a common data model and identified influencing factors for emergency department visits by patients with lung cancer. The predictive model contributes to the efficiency of resource utilization and health care service quality by facilitating the identification and early intervention of high-risk patients. This study demonstrated the possibility of collaborative research among different institutions using the common data model for precision medicine in lung cancer.

## Introduction

Lung cancer is a well-known malignancy that causes severe respiratory symptoms. There were 1.8 million lung cancer–related fatalities and 2.2 million newly diagnosed patients worldwide in 2020 [1]. Patients with lung cancer frequently encounter complex health care challenges, such as unanticipated visits to the emergency department (ED) due to disease progression, treatment-related problems, and comorbidities [2]. Despite advances in medical technology, which have increased the survival rates of patients with lung cancer, ongoing management is still needed after initial oncology treatment due to the diverse characteristics and causes of the disease and the fact that each patient’s disease stage and conditions vary [3].

Patients with lung cancer often experience acute complications or disease progression that may require urgent medical attention [4]. The frequency of ED visits among patients with lung cancer increases with the length of their survival [5]. Prior research has shown that approximately 10% of all cancer-related ED visits are attributable to lung cancer [6]. In addition, compared to patients with other types of cancer, those with lung cancer who visit the ED tend to have a worse prognosis [7]. Shin et al [8] examined the 28-day mortality rate among intubated patients with cancer in the ED. Their findings revealed that patients with lung cancer faced a higher mortality risk compared to those with other types of cancer. Another previous study found that patients with lung cancer had the highest mortality rate (48.1%) within 28 days in contrast to those with other cancers who presented to the ED with septic shock [9]. Further, a comprehensive study conducted on hospitalizations related to sepsis incidence and mortality rates among patients with cancer in the United States revealed that patients with lung cancer had the highest mortality rate [10]. These findings from previous studies demonstrate the poor prognosis for patients with severe lung cancer–related conditions in the ED.

To lower the risk of a poor prognosis, it is of the utmost importance to predict visits to the ED among patients with lung cancer in advance [11]. Nevertheless, the existing literature on this subject is limited, with only a handful of studies identifying the risk factors associated with these visits. Consequently, the lack of comprehensive research in this field hinders clinicians’ ability to intervene in a timely manner. Hong et al [12] developed a machine learning–based model for predicting ED visits in patients who were undergoing radiotherapy or chemoradiotherapy. Sutradhar et al [13] proposed a risk prediction approach for ED visits among patients with cancer using the Edmonton symptom assessment system and conventional statistical analysis and logistic regression methods [13]. Sutradhar and Barbera [14] also developed a machine learning model to predict 7-day ED visits in patients with cancer using the Edmonton symptom assessment system and other clinical information. Previous studies were predominantly conducted among patients with all types of cancer, so they did not identify risk factors specific to those with lung cancer for ED visits.

Therefore, the primary objective of this study was to identify the influential factors for patients with lung cancer that may impact ED visits. This study developed a machine learning–based prediction model to forecast ED visits among patients with lung cancer using data from the Observational Medical Outcomes Partnership (OMOP) common data model (CDM) [15]. Additionally, the significance of various types of clinical information in influencing the decision-making process of the machine learning model was evaluated. The ability to predict ED visits enables health care service providers to identify high-risk patients in advance, allowing for prompt intervention and appropriate management. By intervening expeditiously, clinicians may be able to prevent or mitigate emergency situations, leading to improved patient outcomes. This study contributes to minimizing preventable health deterioration by facilitating early intervention during lung cancer treatment by clinicians.

## Methods

### Study Design and Source of Data

This was a retrospective observational study using electronic health records at Seoul National University Bundang Hospital (SNUBH), a tertiary general hospital in South Korea. The electronic health record data were converted to the OMOP CDM, which is a standardized data format in the observational health data sciences and informatics community. The data set comprised information from visit histories encompassing a broad range of categories, such as diagnoses, medication prescriptions, laboratory test results, performed procedures, and clinical observations.

### Ethical Considerations

This study was conducted with approval and waivers of informed consent or exemptions from the SNUBH institutional review board (no. X-2308-844-903). The CDM data used in this study were deidentified and are securely maintained within an internal network.

### Target Population

**Figure 1:**
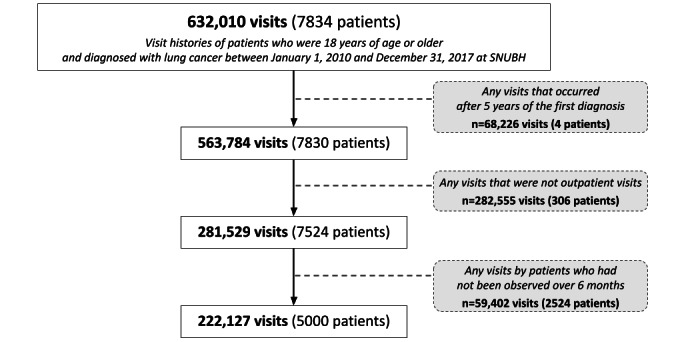
Flowchart of the study participant selection process. SNUBH: Seoul National University Bundang Hospital.

Patients who were 18 years of age or older and diagnosed with lung cancer (International Classification of Diseases, Tenth Revision code C34: malignant neoplasm of bronchus and lung [16]) at least once between 2010 and 2017 were eligible. Figure 1 presents the inclusion and exclusion criteria for the study participants. This study included all outpatient visits within 5 years of the patient’s initial diagnosis of lung cancer. Moreover, since the focus of this study was on health information routinely collected in hospitals based on the CDM, target populations should visit the hospital regularly for disease management. Considering the follow-up period in the treatment guidelines for patients with lung cancer, patients who were unable to follow up for more than 6 months during the study period were excluded.

### Primary Outcome

**Figure 2:**
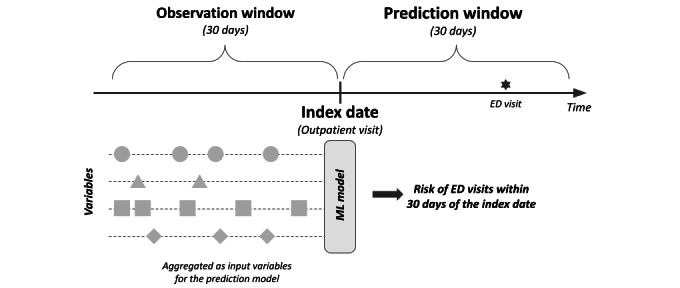
An overview of the observation and prediction windows for the risk prediction task. ED: emergency department; ML: machine learning.

Figure 2 presents an overview of the observation and prediction windows for the risk prediction task. The outpatient visits were defined as the index date. Multiple outpatient visits for the same patient were considered independent index dates. During the 30 days prior to the index date, predictors were extracted and aggregated to be used as input variables for the machine learning models. The primary outcome was the occurrence of ED visits within the 30-day period following the index dates. Since the objective of this study was to predict preventable ED visits that occurred during disease progression or treatment among patients with lung cancer, the primary outcome was defined as ED visits that satisfied specific criteria. Valid outcomes were restricted to ED visits that did not result in a transfer to another hospital.

### Predictors

Data extracted from the CDM over successive time windows, referred to as the observation window, for 30 days prior to the index dates were used for candidate predictors. This included patient demographics, visit histories, clinical information, laboratory results, and vital signs. If patients had multiple records for the same data item during the observation window, the median value was calculated. Detailed information on the selected features and their data sources is presented in Multimedia Appendix 1.

The selected features used to predict the primary outcome were analyzed using statistical methods. This study provided descriptive statistics for continuous variables in the form of median and IQR values, whereas categorical variables were presented as frequencies and respective percentages. The *P* value of each variable was also calculated to explore the probability of a relationship between the selected features for prediction tasks. In the significance test, the Mann-Whitney U test was used when analyzing continuous variables, while the chi-square test was used for categorical data.

### Model Training and Evaluation

This study defined the prediction task for each event date as a binary classification problem. We used 4 different machine learning models—logistic regression, random forest, extreme gradient boosting, and light gradient boosting machine (LGBM)—to discover the model with the highest performance. These models were selected to provide a comparison of models by evaluating the performances of various alternatives, ranging from conventional linear-based approaches to more complex methods, such as ensemble-based models.

The selected features were preprocessed for use as input variables in the machine learning models. Missing values were replaced with their median value, and aberrant observations that were not acceptable from a theoretical perspective were removed based on prior research and the expertise of clinical domain experts to prevent extreme outliers from leading to failure in the prediction task. The entire data set was split into training and testing sets using repeated 7-fold cross-validation, with stratified sampling accounting for the incidence of the primary outcome. Demographic information of the patients, including age, gender, and comorbidities, was also taken into account given that some patients had made multiple outpatient visits, which corresponded to the index dates in this study. By using repeated k-fold cross-validation, the estimated performance of a machine learning model can be enhanced. The cross-validation procedure was iterated 1000 times. Finally, categorical variables were encoded, and continuous variables were normalized.

The following 4 performance metrics were used for model evaluation: area under the receiver operating characteristic curve (AUROC), area under the precision-recall curve (PRAUC), sensitivity, and specificity. The evaluation results were reported as the average value encompassing all folds from all iterations, and the 95% CI was estimated. This approach ensures a more accurate estimate of the true unknown underlying mean performance of the model on the data set than using the standard error. Using the Shapley additive explanations (SHAP) value from the best performing model, the significance of the features was also analyzed to identify risk factors for ED visits by patients with lung cancer [17]. All the experiments were performed using the Python 3.7.6 environment (Python Software Foundation).

## Results

### Baseline Characteristics

A total of 222,127 outpatient visits occurred for 5,000 patients. Table 1 presents the baseline characteristics of the target population. The median age was 67.23 (IQR 60.54-75.57), and 3812 (76.24%) patients were older than 60 years. Men accounted for 64.14% (n=3322) of the participants, which was slightly greater than the number of women (n=1678). According to the Charlson comorbidity index (CCI) [18], more than 66.4% (n=3320) of participants did not have any comorbidities before being diagnosed with lung cancer. There were 1212 (24.24%) patients who had a history of smoking, while the others had no smoking history or their history was unknown. About half (n=2827, 56.54%) of the patients lived in the metropolitan area near the hospital.

**Table 1:** Baseline characteristics of the selected participants (n=5000).

Characteristic		Value
Age (years), median (IQR)		67.23 (60.54-75.57)
**Age group (years), n (%)**		
	18-49	376 (7.52)
	50-59	812 (16.24)
	60-69	1511 (30.22)
	≥70	2301 (46.02)
**Gender, n (%)**		
	Men	3322 (64.14)
	Women	1678 (35.86)
Body mass index, median (IQR)		22.90 (20.59-24.97)
Charlson comorbidity index, median (IQR)		2.95 (2.00-3.00)
**Charlson comorbidity index group, n (%)**		
	0	3320 (66.4)
	1-2	982 (19.64)
	3-4	299 (5.98)
	≥5	399 (7.98)
**Smoking history, n (%)**		
	Yes	1212 (24.24)
	No	957 (19.14)
	Unknown	4600 (56.62)
**Residence, n (%)**		
	Greater Seoul	2827 (56.54)
	Other^a^	1071 (21.42)
	Unknown	1102 (22.04)

^a^Other areas of residence included Gangwon-do, Chungcheongbuk-do, Chungcheongnam-do, Gyeongsangbuk-do, Gyeongsangnam-do, Jeollabuk-do, Jeollanam-do, Jeju-do, Daejeon, Sejong-si, Daegu, Ulsan, Busan, and Gwangju.

There were 8192 visits to the ED, and 2790 patients (55.80% of total patients) had ED visits during the course of their disease. The results of an explanatory data analysis of ED visits by the target population are displayed in Table 2. Of all the visits, 81.33% (n=6663) were from home, while the rest were from outpatient visits or other institutions, including transfers from hospitals, independent clinics, and inpatient care units. Most ED visits occurred between 7 AM and 10 PM. The median length of time spent in the ED was approximately 13.64 (IQR 2.98-15.84) hours. More than half (n=4956) of the patients were discharged home, while the remaining patients were hospitalized or died in hospital. Of all ED visits, 38.32% (n=3139) resulted in hospitalization, and 1.18% (n=97) resulted in death. The most frequent causes of visits to the ED were neoplasms, including malignant neoplasms of the bronchus and lung and secondary malignant neoplasms of other sites. The other primary causes were diseases of the respiratory system. Patients with symptoms, signs, and abnormal clinical and laboratory findings not elsewhere classified primarily visited the ED for the following reasons: fever of other and unknown origin, hemorrhage from respiratory passages, abnormalities of breathing, pain in the throat and chest, and abdominal and pelvic pain.

**Table 2:** Explanatory data analysis results of emergency department (ED) visits (n=8192) by selected patients.

Feature		Value
Age (years), median (IQR)		67.10 (60.26-75.50)
**Gender, n (%)**		
	Men	5502 (67.16)
	Women	2690 (32.84)
**Sourced from, n (%)**		
	Home	6663 (81.33)
	Outpatient visits	697 (8.51)
	Other institutions	832 (10.16)
**Discharged to, n (%)**		
	Home	4956 (60.50)
	Hospitalization	3139 (38.32)
	Death	97 (1.18)
**Visit time of day, n (%)**		
	7AM to 3 PM	4842 (59.11)
	3 PM to 10 PM	2374 (28.98)
	10 PM to 7 AM	976 (11.91)
Time spent in ED, median (IQR)		13.64 (2.98-15.84)
**Primary diagnosis (ICD-10^a^ codes), n (%)**		
	Neoplasms (C00-D48)	4242 (51.78)
	Diseases of the respiratory system (J00-J99)	967 (11.8)
	Symptoms, signs, and abnormal clinical and laboratory findings not elsewhere classified (R00-R99)	886 (10.82)
	Diseases of the digestive system (K00-K93)	361 (4.41)
	Diseases of the circulatory system (I00-I99)	307 (3.75)
	Other	1429 (17.44)

^a^ICD-10: International Classification of Diseases, Tenth Revision.

The complete list of descriptive statistics (N=222,127) of the selected features used as input variables for the predictive model are displayed in Multimedia Appendix 2. The median value of elapsed days since the first diagnosis of lung cancer was about 333 (IQR 103.60-810.42) days. Chemotherapy was administered at 39.27% (n=87,221) of visits, compared to 16.47% (n=36,575) for radiation therapy and 2.32% (n=5159) for lung cancer–related surgery. Analgesics were administered at 27.35% (n=60,744) of visits, and the use of antibacterials for systemic use accounted for 18.83% (n=41,825) of visits. The results of blood tests revealed a median value of 6.27 (IQR 4.87-8.06) for leukocytes, 237.00 (IQR 188.00-296.00) for platelets, 61.90 (IQR 53.50-70.05) for neutrophils, and 12.10 (IQR 10.80-13.30) for hemoglobin. The shock index, which refers to the ratio of the heart rate to systolic blood pressure, was calculated from the collected vital signs and showed a median value of 0.67 (IQR 0.59-0.78).

### Performance Evaluation Results of the Machine Learning Models

The performance evaluation results from the machine learning models are presented in Table 3. The overall AUROC score was ≥0.70 in all models, ranging from 0.70 to 0.73. The optimal prediction threshold was defined using the precision-recall curve since the data set had an imbalanced class distribution. The highest PRAUC score was 0.24 in the LGBM model.

**Table 3:** Performance comparison of the machine learning models.

Model	Sensitivity (95% CI)	Specificity (95% CI)	AUROC^a^ (95% CI)	PRAUC^b^ (95% CI)
LR^c^	0.76 (0.7554-0.7581)	0.54 (0.5369-0.5398)	0.71 (0.7054-0.7065)	0.22 (0.2191-0.2205)
RF^d^	0.74 (0.7360-0.7395)	0.57 (0.5634-0.5667)	0.71 (0.7086-0.7097)	0.22 (0.2205-0.2221)
XGB^e^	0.77 (0.7729-0.7759)	0.51 (0.5080-0.5112)	0.70 (0.7022-0.7033)	0.21 (0.2134-0.2145)
LGBM^f^	0.76 (0.7575-0.7605)	0.58 (0.5752-0.5780)	0.73 (0.7312-0.7323)	0.24 (0.2360-0.2374)

^a^AUROC: area under the receiver operating characteristic curve.

^b^PRAUC: area under the precision-recall curve.

^c^LR: logistic regression.

^d^RF: random forest.

^e^XGB: extreme gradient boosting.

^f^LGBM: light gradient boosting machine.

**Figure 3:**
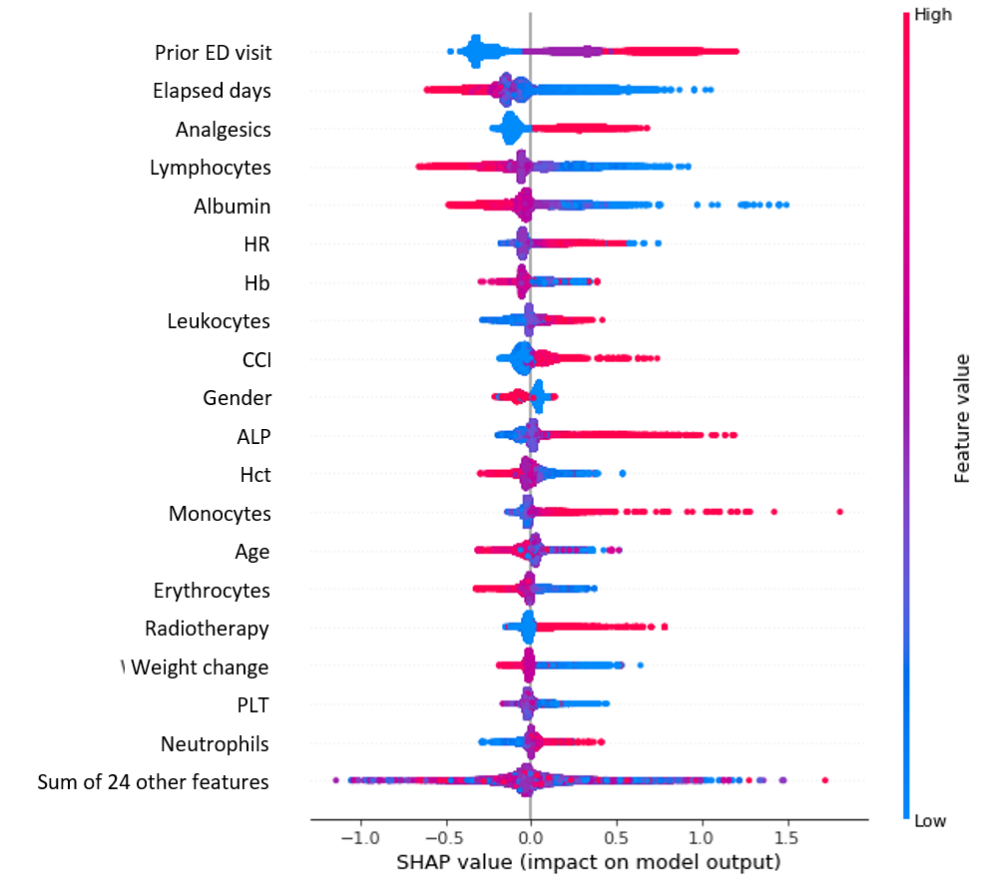
Summary plots for SHAP values in the light gradient boosting machine model. ALP: alkaline phosphatase; CCI: Charlson comorbidity index; ED: emergency department; Hb: hemoglobin; Hct: hematocrit; HR: heart rate; PLT: platelets; SHAP: Shapley additive explanations.

The SHAP values for the highest-performing model, the LGBM, are depicted in Figure 3. The key influencing features for predicting the risk of ED visits in the LGBM model were identified as recent ED visits, elapsed days since the initial diagnosis for lung cancer, the use of analgesics, and lymphocyte and albumin levels. The SHAP value quantifies the influence of a specific feature on the predictions; consequently, a comprehensive interpretation can be obtained by calculating the SHAP values for the model’s output. As shown in Figure 3, the number of ED visits, CCI, administration of analgesics, and radiotherapy, as well as high values of leukocytes, alkaline phosphatase, monocytes, and neutrophils, increased the possibility of visits to the ED. In contrast, the combined presence of low values of lymphocytes, albumin, hemoglobin, and hematocrit indicated an increased likelihood of visiting the ED.

## Discussion

This study developed a predictive model for ED visits among patients with lung cancer using machine learning and CDM data. Predicting ED visits among patients with lung cancer has numerous important implications. First, the ED visit prediction model is able to facilitate care coordination and patient management. As shown in Table 2, over 38% of all visits resulted in hospitalization. These results were marginally lower than the findings in prior literature, which indicated that 54.5% of ED visits resulted in hospitalization or death [19]. This may be due to the SNUBH-specific aspect that patients with cancer are treated via outpatient visits, and patients who were normally discharged might return to other inpatient care units or independent clinics after emergency treatments. If patients at risk could be identified during a previous scheduled session, many of these visits might be avoided. Moreover, it would be possible to perform elective procedures to manage patients at imminent risk of requiring ED visits. There have been several prior studies on predicting ED visits [20-22]; however, to our knowledge, there have been no studies predicting ED visits applicable to outpatient visits by patients with lung cancer. In addition, previous studies mainly focused on patient-reported outcomes, whereas this study mainly used clinical information, such as laboratory test results, as input variables for the prediction model. The clinical information–based machine learning model enhances the clinical use of the predictive model since patients with lung cancer continuously visit the hospital during their disease or after treatment for follow-up [23].

The identification of risk factors associated with ED visits among patients with lung cancer is another significant finding of this study. The risk factors were identified by analyzing the importance of features that influenced the decision-making of machine learning models, as shown in Figure 3, and the identified variables had a tendency to match theoretical knowledge found in the medical domain. Prior ED use was known to be a significant predictor of future ED visits for all types of patients with cancer [12]. Patients with cancer who had recently visited the ED more frequently may experience more severe symptoms, complications, or comorbidities requiring immediate medical care, thereby increasing their likelihood of future ED visits. The number of elapsed days since the initial lung cancer diagnosis was inversely proportional to the likelihood of visiting the ED. Given that cancer-related treatments, such as surgery, chemotherapy, and radiotherapy, are typically administered within a year of diagnosis, acute diseases and transient side effects of the cancer-related treatment can result in emergency situations [24]. Similarly, the use of analgesics indicates that patients were suffering from severe pain and distress, which may have prompted visits to the ED for pain management. Several clinical pieces of information, such as laboratory test results routinely collected during cancer treatment and follow-up monitoring, were demonstrated to be useful predictors of ED visits in patients with lung cancer. Lower lymphocyte counts may indicate decreased immune function or increased vulnerability to infections, and higher leukocyte, monocyte, and neutrophil counts may be associated with inflammation; both of these abnormal values may lead to visits to the ED. Lower levels of hemoglobin, hematocrit, and erythrocyte counts may be signs of anemia, which may cause fatigue and result in visits to the ED. Significant weight loss and lower albumin levels may be associated with disease progression or malnutrition, increasing the risk of complications that may require ED visits. Similarly, a high CCI indicated the presence of multiple comorbid conditions. Elevated alkaline phosphatase levels may indicate liver or bone involvement, and decreased platelet counts may indicate thrombocytopenia, necessitating evaluation and treatment in the ED. While radiotherapy is a treatment for lung cancer, it can cause side effects and complications, such as radiation pneumonitis, that may necessitate visits to the ED. Thus, close monitoring of laboratory test results in patients with lung cancer receiving treatments or routine follow-up is necessary to prevent ED visits.

This study has several limitations. First, this study was conducted at a single institution in South Korea, a tertiary-level general hospital. Thus, regional bias may have resulted in reduced generalizability. Nonetheless, this study demonstrated the possibility of conducting observational cancer research with CDM data. Developing a predictive model using data from a single institution may lead to biased results based on regional or institution-specific characteristics, making it difficult to generalize; therefore, external validation is essential for clinical applications. Due to the heterogeneity of the format for managing medical records across institutions as well as privacy concerns, it is difficult for researchers to share data; even a collaborative study with other institutions requires immense effort, time, and resources. Ahmadi et al [25] reported that the OMOP CDM has the potential to facilitate international collaborative analyses, which is a crucial element of cancer precision medicine. Since our prediction model was developed using OMOP CDM data, it is expected that external validation will be possible for other institutions that have oncology CDM data through the observational health data sciences and informatics data network in the future. The data set used in this study is administered in accordance with the OMOP CDM, allowing different institutions to conduct collaborative research using a distributed research network.

Second, there were the inherent performance limitations imposed on the machine learning models. The primary outcome defined in this study was derived from all ED visits, only excluding the visits that resulted in patients being transferred to other institutions; therefore, there may have been non–cancer-related reasons for ED visits, such as fractures, injuries from traffic accidents, or wounds from animal attacks. In addition, a paucity of information may be one of the reasons for the results of unfavorable effects on the prediction model outcome. Staging or subtypes of lung cancer represent primary information for predicting the prognosis of patients; however, this information was not collected in our data source thus far. The acquisition of additional information could contribute to the enhancement of the predictive model’s performance; therefore, the establishment of extended lung cancer–specific data sets in the CDM is needed for more precise prediction. Nevertheless, the identified risk factors for ED visits could be used in the future as an avenue for the proactive management of patients with lung cancer during treatment.

Lastly, research bias should be taken into account when interpreting the results. This study imputed missing values with the median value during the data processing phase, which could potentially have introduced bias into the analysis. However, the characteristics of clinical data are such that the lack of even a solitary test result frequently results in a substantial amount of missing data among other relevant data, indicating the presence of a clear-cut pattern. In such situations, it was noted that median imputation demonstrated performance levels that were comparable to those of more complex algorithms. Furthermore, it offered the advantage of being readily executable, thereby enhancing its efficiency in terms of time and resources. Additionally, it is critical to acknowledge that while removing outliers is a standard procedure for enhancing data quality, extreme values may have clinical ramifications due to the unique attributes of ED visits among patients diagnosed with lung cancer.

In summary, this study developed a machine learning model to predict ED visits, with a specific focus on patients with lung cancer, and identified influential factors that exerted a significant impact on the model output. Continuous monitoring of the identified influencing clinical features will allow health care providers to efficiently allocate resources, ensuring that patients with a high likelihood of requiring preventive care receive prompt attention. By identifying patients at risk, health care service providers can initiate targeted interventions, such as closer monitoring, treatment plan adjustments, and timely referrals to the most appropriate specialists. The predictive model for ED visits by patients with lung cancer developed in this study not only enhances patient outcomes but also optimizes resource utilization, reducing strain on the ED and minimizing the burden on the medical staff. Ultimately, this proactive approach contributes to preventing or mitigating emergency situations, resulting in an improved quality of life for patients with lung cancer and possibly reducing the need for hospitalization or more invasive interventions.

## Appendix

Multimedia Appendix 1 A list of selected features.

Multimedia Appendix 2 Descriptive statistics of the selected features.

## Abbreviations

AUROC: area under the receiver operating characteristic curve
CCI: Charlson comorbidity index
CDM: common data model
ED: emergency department
LGBM: light gradient boosting machine
OMOP: observational medical outcomes partnership
PRAUC: area under the precision-recall curve
SHAP: Shapley additive explanations
SNUBH: Seoul National University Bundang Hospital

## References

[1] Sung H, Ferlay J, Siegel R, Laversanne M, Soerjomataram I, Jemal A, Bray F (2021). Global cancer statistics 2020: GLOBOCAN estimates of incidence and mortality worldwide for 36 cancers in 185 countries. CA Cancer J Clin.

[2] Gallaway MS, Idaikkadar N, Tai E, Momin B, Rohan EA, Townsend J, Puckett M, Stewart SL (2021). Emergency department visits among people with cancer: frequency, symptoms, and characteristics. J Am Coll Emerg Physicians Open.

[3] Barta JA, Powell CA, Wisnivesky JP (2019). Global epidemiology of lung cancer. Ann Glob Health.

[4] Panattoni L, Fedorenko C, Greenwood-Hickman MA, Kreizenbeck K, Walker JR, Martins R, Eaton KD, Rieke JW, Conklin T, Smith B, Lyman G, Ramsey SD (2018). Characterizing potentially preventable cancer- and chronic disease–related emergency department use in the year after treatment initiation: a regional study. J Oncol Pract.

[5] Cronin KA, Lake AJ, Scott S, Sherman RL, Noone A, Howlader N, Henley SJ, Anderson RN, Firth AU, Ma J, Kohler BA, Jemal A (2018). Annual report to the nation on the status of cancer, part I: national cancer statistics. Cancer.

[6] Walder JR, Faiz SA, Sandoval M (2023). Lung cancer in the emergency department. Emerg Cancer Care.

[7] Kotajima F, Kobayashi K, Sakaguchi H, Nemoto M (2014). Lung cancer patients frequently visit the emergency room for cancer-related and -unrelated issues. Mol Clin Oncol.

[8] Shin SH, Lee H, Kang HK, Park JH (2019). Twenty-eight-day mortality in lung cancer patients with metastasis who initiated mechanical ventilation in the emergency department. Sci Rep.

[9] Kim Y, Kang J, Kim M, Ryoo SM, Kang GH, Shin TG, Park YS, Choi S, Kwon WY, Chung SP, Kim WY (2020). Development and validation of the VitaL CLASS score to predict mortality in stage IV solid cancer patients with septic shock in the emergency department: a multi-center, prospective cohort study. BMC Med.

[10] Liu M, Bakow B, Hsu T, Chen J, Su K, Asiedu E, Hsu WT, Lee CC (2021). Temporal trends in sepsis incidence and mortality in patients with cancer in the US population. Am J Crit Care.

[11] Alandonisi M, Al-Malki H, Bahaj W, Alghanmi H (2023). Characteristics of emergency visits among lung cancer patients in comprehensive cancer center and impact of palliative referral. Cureus.

[12] Hong JC, Niedzwiecki D, Palta M, Tenenbaum JD (2018). Predicting emergency visits and hospital admissions during radiation and chemoradiation: an internally validated pretreatment machine learning algorithm. JCO Clinical Cancer Informatics.

[13] Sutradhar R, Rostami M, Barbera L (2019). Patient-reported symptoms improve performance of risk prediction models for emergency department visits among patients with cancer: a population-wide study in Ontario using administrative data. J Pain Symptom Manage.

[14] Sutradhar R, Barbera L (2020). Comparing an artificial neural network to logistic regression for predicting ED visit risk among patients with cancer: a population-based cohort study. J Pain Symptom Manage.

[15] Overhage JM, Ryan PB, Reich CG, Hartzema AG, Stang PE (2012). Validation of a common data model for active safety surveillance research. J Am Med Inform Assoc.

[16] ICD-10 version:2019. World Health Organization.

[17] Lundberg SM, Lee SI (2017). A unified approach to interpreting model predictions. http://papers.nips.cc/paper/7062-a-unified-approach-to-interpreting-model-predictions.pdf.

[18] Roffman CE, Buchanan J, Allison GT (2016). Charlson comorbidities index. J Physiother.

[19] Lee SY, Ro YS, Shin SD, Moon S (2021). Epidemiologic trends in cancer-related emergency department utilization in Korea from 2015 to 2019. Sci Rep.

[20] Katzan IL, Thompson N, Schuster A, Wisco D, Lapin B (2021). Patient‐reported outcomes predict future emergency department visits and hospital admissions in patients with stroke. J Am Heart Assoc.

[21] Chen R, Cheng K, Lin Y, Chang I, Tsai C (2021). Predicting unscheduled emergency department return visits among older adults: population-based retrospective study. JMIR Med Inform.

[22] Gao K, Pellerin G, Kaminsky L (2018). Predicting 30-day emergency department revisits. Am J Manag Care.

[23] Owusuaa C, van der Padt-Pruijsten A, Drooger JC, Heijns JB, Dietvorst A, Janssens-van Vliet ECJ, Nieboer D, Aerts JGJV, van der Heide A, van der Rijt CCD (2022). Development of a clinical prediction model for 1-year mortality in patients with advanced cancer. JAMA Netw Open.

[24] Bironzo P, Di Maio M (2018). A review of guidelines for lung cancer. J Thorac Dis.

[25] Ahmadi N, Peng Y, Wolfien M, Zoch M, Sedlmayr M (2022). OMOP CDM can facilitate data-driven studies for cancer prediction: a systematic review. Int J Mol Sci.

