# Supplementary material for: Risk Prediction of Emergency Department Visits in Patients With Lung Cancer Using Machine Learning: Retrospective Observational Study
Source: JMIR Med Inform. 2023 Dec 6;11:e53058. doi: 10.2196/53058 (PMC10733827; doi:10.2196/53058)
Supplement: Multimedia Appendix 1 [file medinform_v11i1e53058_app1.pdf]

Multimedia Appendix 1. A list of selected features

Features	Description	Sampling time	Unit	CDM Table
**Demographics**				
Age	Age	index date	year	PERSON
Sex	Male | Female	index date	-	PERSON
CCI	Charlson Comorbidity Index	index date	-	CONDITION_OCCURRENCE
Smoking history	Tobacco use and exposure	index date	-	OBSERVATION
Weight change	The ratio of the median weight over the last 30 days to the previous year	within a year before the index date	%	MEASUREMENT
**Visit history**				
Elapsed days	Days from first diagnosis of lung cancer	index date	day	VISIT_OCCURRENCE
Recent ED visits	Number of ED visits in the previous year	within a year before the index date	-	VISIT_OCCURRENCE
Recent hospitalizations	Number of hospitalizations in the previous year	within a year before the index date	-	VISIT_OCCURRENCE
**Clinical information**				
Lung cancer-related surgery	Excision, Segmental resection, Lobectomy, Pneumonectomy	within 30 days before the index date	-	PROCEDURE_OCCURRENCE
Antineoplastic agents	ATC code L01	within 30 days before the index date	-	DRUG_ERA
Antibacterials for systemic use	ATC code J01	within 30 days before the index date	-	DRUG_ERA
Analgesics	ATC code N02	within 30 days before the index date	-	DRUG_ERA
**Laboratory Test**				

Leukocytes	Leukocytes [#/volume] in Blood by Automated count	within 30 days before the index date	x10^3/㎕	MEASUREMENT
Hb	Hemoglobin [Mass/volume] in Blood	within 30 days before the index date	g/㎗	MEASUREMENT
PLT	Platelets [#/volume] in Blood by Automated count	within 30 days before the index date	x10^3/㎕	MEASUREMENT
Neutrophil	Segmented neutrophils/100 leukocytes in Blood by Automated count	within 30 days before the index date	%	MEASUREMENT
Hct	Hematocrit [Volume Fraction] of Blood by Automated count	within 30 days before the index date	%	MEASUREMENT
Erythrocytes	Erythrocytes [#/volume] in Blood by Automated count	within 30 days before the index date	x10^6/㎕	MEASUREMENT
Lymphocytes	Lymphocytes/100 leukocytes in Blood by Automated count	within 30 days before the index date	%	MEASUREMENT
Eosinophils	Eosinophils/100 leukocytes in Blood by Automated count	within 30 days before the index date	%	MEASUREMENT
Basophils	Basophils/100 leukocytes in Blood by Automated count	within 30 days before the index date	%	MEASUREMENT
Monocytes	Monocytes/100 leukocytes in Blood by Automated count	within 30 days before the index date	%	MEASUREMENT
MCV	MCV [Entitic volume]	within 30 days before the index date	fL	MEASUREMENT
MCH	MCV [Entitic volume]	within 30 days before the index date	pg	MEASUREMENT
MCHC	MCHC [Mass/volume]	within 30 days before the index date	g/㎗	MEASUREMENT

Cr	Creatinine [Mass/volume] in Serum or Plasma	within 30 days before the index date	㎎/㎗	MEASUREMENT
Albumin	Albumin [Mass/volume] in Serum or Plasma	within 30 days before the index date	g/㎗	MEASUREMENT
BUN	Urea nitrogen [Mass/volume] in Serum or Plasma	within 30 days before the index date	㎎/㎗	MEASUREMENT
Calcium	Calcium [Mass/volume] in Serum or Plasma	within 30 days before the index date	㎎/㎗	MEASUREMENT
Phosphate	Phosphate [Mass/volume] in Serum or Plasma	within 30 days before the index date	㎎/㎗	MEASUREMENT
Bilirubin	Bilirubin.total [Mass/volume] in Serum or Plasma	within 30 days before the index date	㎎/㎗	MEASUREMENT
Protein	Protein [Mass/volume] in Serum or Plasma	within 30 days before the index date	g/㎗	MEASUREMENT
ALP	Alkaline phosphatase [Enzymatic activity/volume] in Serum or Plasma	within 30 days before the index date	IU/L	MEASUREMENT
Cholesterol	Cholesterol [Mass/volume] in Serum or Plasma	within 30 days before the index date	㎎/㎗	MEASUREMENT
Glucose	Glucose [Mass/volume] in Serum or Plasma	within 30 days before the index date	㎎/㎗	MEASUREMENT
eGFR	CKD-EPI Estimated Glomerular Filteration Rate, Creatinine-based	within 30 days before the index date	㎖/min	MEASUREMENT
**Vital Signs**				
HR	Heart rate	within 30 days before the index date	beats/min	MEASUREMENT
RR	Respiratory rate	within 30 days before	breaths/min	MEASUREMENT

		the index date		
SBP	Systolic blood pressure	within 30 days before the index date	mmHg	MEASUREMENT
DBP	Diastolic blood pressure	within 30 days before the index date	mmHg	MEASUREMENT
O2 saturation	Oxygen saturation in Arterial blood by Pulse oximetry	within 30 days before the index date	%	MEASUREMENT
BT	Body temperature	within 30 days before the index date	℃	MEASUREMENT
Shock index	HR / SBP	within 30 days before the index date	-	MEASUREMENT

CDM=Common data model; IQR=Interquartile range; CCI=Charlson Comorbidity Index; BMI=Body Mass Index; Hb=Hemoglogin; PLT=Platelets; Hct=Hematocrit; MCV=Mean Corpuscular Volume; MCH=Mean Corpuscular Hemoglobin; MCHC=Mean Corpuscular Hemoglobin Concentration; Cr=Creatinine; BUN=Urea nitrogen; ALP=Alkaline Phosphatase; eGFR=Estimated Glomerular Filteration Rate; CKD=Chronic kidney disease; EPI=Epidemiology collaboration; HR=Heart Rate; RR=Respiratory Rate; SBP=Systolic Blood Pressure; DBP=Diastolic Blood Pressure; BT=Body Temperature;
