# Supplementary material for: Risk Prediction of Emergency Department Visits in Patients With Lung Cancer Using Machine Learning: Retrospective Observational Study
Source: JMIR Med Inform. 2023 Dec 6;11:e53058. doi: 10.2196/53058 (PMC10733827; doi:10.2196/53058)
Supplement: Multimedia Appendix 2 [file medinform_v11i1e53058_app2.docx]

**Multimedia Appendix 2.** Descriptive statistics of the selected features

| **Characteristic** | **Missing** | **Total (n=222,127)** | **Class 0  (n=198,547)** | **Class 1 (n=23,580)** | **p-Value** |
| --- | --- | --- | --- | --- | --- |
| **Demographics** |  |  |  |  |  |
| Age, median [Q1, Q3] | 0 | 67.49 [59.69, 74.46] | 67.50 [59.71, 74.50] | 67.36 [59.61. 74.11] | 0.002 |
| Sex, n (%) | 0 |  |  |  | <0.001 |
| Male |  | 142,116 (63.98) | 125,470 (63.19) | 166,46 (70.59) |  |
| Female |  | 80,011 (36.02) | 73,077 (36.81) | 6,934 (29.41) |  |
| Weight change, median [Q1, Q3] | 102,100 | 0.00 [-1.71, 0.89] | 0.00 [-1.58, 0.95] | -0.21 [-2.80, 0.42] | <0.001 |
| CCI, median [Q1, Q3] | 0 | 3.00 [2.00, 8.00] | 3.00 [2.00, 8.00] | 4.00 [2.00, 8.009 | <0.001 |
| Smoking history, n (%) | 17,329 |  |  |  | <0.001 |
| Yes |  | 116,633 (52.51) | 103,263 (56.45) | 13,370 (61.15) |  |
| No |  | 88,165 (39.69) | 79,670 (43.55) | 8,495 (38.85) |  |
| **Visit History** |  |  |  |  |  |
| Elapsed days, median [Q1, Q3] | 0 | 332.58 [103.60, 810.42] | 347.66 [106.58, 855.47] | 226.62 [78.37. 587.46] | <0.001 |
| Recent ED visits, median [Q1, Q3] | 0 | 0.00 [0.00, 1.00] | 0.00 [0.00, 1.00] | 1.00 [0.00, 2.00] | <0.001 |
| **Clinical information** |  |  |  |  |  |
| Lung cancer-related surgery, n (%) | 0 |  |  |  | 0.040 |
| Yes |  | 5,159 (2.32) | 4,566 (2.30) | 593 (2.51) |  |
| No |  | 216,968 (97.68) | 193,981 (97.70) | 22,987 (97.49) |  |
| Radiotherapy | 0 |  |  |  | <0.001 |
| Yes |  | 36,575 (16.47) | 31,120 (15.67) | 5,455 (23.13) |  |
| No |  | 185,552 (83.53) | 167,427 (84.33) | 18,125 (76.87) |  |
| Antineoplasitc agents, n (%) | 0 |  |  |  | <0.001 |
| Yes |  | 87,221 (39.27) | 76,750 (38.66) | 10,471 (44.41) |  |
| No |  | 134,906 (60.73) | 121,797 (61.34) | 13,109 (55.59 |  |
| Antibacterials for systemic use, n (%) | 0 |  |  |  | <0.001 |
| Yes |  | 41,825 (18.83) | 34,993 (17.62) | 6,832 (28.97) |  |
| No |  | 180,302 (81.17) | 163,554 (82.38) | 16,748 (71.03) |  |
| Analgesics, n (%) | 0 |  |  |  | <0.001 |
| Yes |  | 60,744 (27.35) | 50,414 (25.39) | 10,330 (43.81) |  |
| No |  | 161,383 (72.65) | 148,133 (74.61) | 13,250 (56.190 |  |
| **Laboratory Test** |  |  |  |  |  |
| Leukocytes, median [Q1, Q3] | 43,411 | 6.27 [4.87, 8.06] | 6.20 [4.83, 7.93] | 6.85 [5.17, 9.05] | <0.001 |
| Hb, median [Q1, Q3] | 43,411 | 12.10 [10.80, 13.30] | 12.20 [10.90, 13.40] | 11.40 10.15, 12.80] | <0.001 |
| PLT, median [Q1, Q3] | 43,411 | 237.00 [188.00, 296.00] | 236.00 [188.00, 294.00] | 242.00 [184.50, 313.00] | <0.001 |
| Neutrophil, median [Q1, Q3] | 44,510 | 61.90 [53.50, 70.05] | 61.25 [53.00, 69.35] | 66.70 [57.90, 74.50] | <0.001 |
| Hct, median [Q1, Q3] | 43,411 | 36.40 [32.55, 39.90] | 36.70 [32.90, 40.10] | 34.25 [30.60, 38.25] | <0.001 |
| Erythrocytes, median [Q1, Q3] | 43,411 | 3.93 [3.49, 4.34] | 3.96 [3.52, 4.36] | 3.72 [3.29, 4.17] | <0.001 |
| Lymphocytes, median [Q1, Q3] | 44,510 | 23.10 [18.10, 32.90] | 25.80 [18.75, 33.40] | 20.30 [14.10, 27.90] | <0.001 |
| Eosinophils, median [Q1, Q3] | 44,762 | 1.70 [0.85, 3.10] | 1.80 [0.90, 3.20] | 1.40 [0.65, 2.75] | <0.001 |
| Basophils, median [Q1, Q3] | 45,167 | 0.40 [0.30, 0.60] | 0.40 [0.30, 0.60] | 0.40 [0.20, 60] | <0.001 |
| Monocytes, median [Q1, Q3] | 44,513 | 8.65 [6.90, 11.10] | 8.60 [6.90, 11.00] | 8.80 [6.85, 11.45] | <0.001 |
| MCV, median [Q1, Q3] | 43,411 | 92.80 [89.50, 96.20] | 92.90 [89.60, 96.20] | 92.30 [88.90, 95.85] | <0.001 |
| MCH, median [Q1, Q3] | 43,411 | 31.00 [29.75, 32.30] | 31.00 [29.80, 32.30] | 30.90 [29.50, 32.20] | <0.001 |
| MCHC, median [Q1, Q3] | 43,411 | 33.30 [32.65, 34.00] | 33.30 [32.65, 34.00] | 33.30 [32.60, 34.00] | 0.189 |
| Cr, median [Q1, Q3] | 47,646 | 0.83 [0.69, 1.01] | 0.83 [0.69, 1.01] | 0.84 [0.69, 1.03] | <0.001 |
| Albumin, median [Q1, Q3] | 51,326 | 4.00 [3.65, 4.20] | 4.00 [3.70, 4.25] | 3.75 [3.40, 4.10] | <0.001 |
| BUN, median [Q1, Q3] | 62,827 | 15.00 [12.00, 19.50] | 15.00 [12.00, 19.00] | 16.00 [12.00, 20.00] | <0.001 |
| Calcium, median [Q1, Q3] | 63,165 | 8.90 [8.60, 9.20] | 8.90 [8.60, 9.20] | 8.80 [8.50, 9.10] | <0.001 |
| Phosphate, median [Q1, Q3] | 63,174 | 3.40 [3.05, 3.80] | 3.40 [3.05, 3.80] | 3.35 [3.00, 3.70] | <0.001 |
| Bilirubin, median [Q1, Q3] | 51,368 | 0.51 [0.40, 0.70] | 0.52 [0.40, 0.70] | 0.50 [0.40, 0.70] | <0.001 |
| Protein, median [Q1, Q3] | 51,422 | 6.80 [6.40, 7.20] | 6.85 [6.50, 7.20] | 6.70 [6.20, 7.10] | <0.001 |
| ALP, median [Q1, Q3] | 51,399 | 83.00 [68.00, 105.00] | 83.00 [67.50, 104.00] | 88.75 [70.50, 117.00] | <0.001 |
| Cholesterol, median [Q1, Q3] | 52,780 | 172.00 [145.00, 200.00] | 173.00 [146.00, 200.50] | 164.00 [136.50, 194.00] | <0.001 |
| Glucose, median [Q1, Q3] | 65,060 | 110.00 [96.00, 137.00] | 109.00 [96.00, 135.00] | 117.00 [99.50, 150.00] | <0.001 |
| eGFR, median [Q1, Q3] | 47,646 | 92.20 [72.90, 104.20] | 92.20 [73.10, 104.10] | 92.10 [71.50, 104.40] | 0.367 |
| **Vital Signs** |  |  |  |  |  |
| HR, median [Q1, Q3] | 85,546 | 82.50 [73.00, 93.00] | 82.00 [73.00, 92.00] | 86.50 [76.00, 97.50] | <0.001 |
| RR, median [Q1, Q3] | 136,477 | 18.00 [18.00, 20.00] | 18.00 [18.00, 20.00] | 18.00 [18.00, 20.00] | <0.001 |
| SBP, median [Q1, Q3] | 84,131 | 122.00 [113.00, 132.50] | 122.00 [113.00, 133.00] | 121.00 [112.00, 131.50] | <0.001 |
| DBP, median [Q1, Q3] | 84,125 | 72.50 [66.50, 79.00] | 72.50 [66.50, 79.00] | 72.00 [66.00, 78.00] | <0.001 |
| O2 saturation, median [Q1, Q3] | 167,807 | 97.00 [96.00, 98.00] | 97.00 [96.00, 98.50] | 97.00 [96.00, 98.00] | <0.001 |
| BT, median [Q1, Q3] | 126,125 | 36.00 [36.40, 36.80] | 36.55 [36.40, 36.75] | 36.60 [36.40, 36.80] | <0.001 |
| Shock index, median [Q1, Q3] | 85,593 | 0.67 [0.59, 0.78] | 0.67 [0.58, 0.77] | 0.71 [0.61, 0.82] | <0.001 |

IQR=Interquartile range; CCI=Charlson Comorbidity Index; BMI=Body Mass Index; Hb=Hemoglogin; PLT=Platelets; Hct=Hematocrit; MCV=Mean Corpuscular Volume; MCH=Mean Corpuscular Hemoglobin; MCHC=Mean Corpuscular Hemoglobin Concentration; Cr=Creatinine; BUN=Urea nitrogen; ALP=Alkaline Phosphatase; eGFR=Estimated Glomerular Filteration Rate; HR=Heart Rate; RR=Respiratory Rate; SBP=Systolic Blood Pressure; DBP=Diastolic Blood Pressure; BT=Body Temperature;
